# Melioidosis Presenting As a Chronic Splenic Abscess: A Diagnostic Challenge

**DOI:** 10.7759/cureus.107936

**Published:** 2026-04-29

**Authors:** Sai Prathyusha Tatineni, Sivaprakash Varadan, Vaishnavi Sekar, R B Sudagar Singh

**Affiliations:** 1 Internal Medicine, Sri Ramachandra Institute of Higher Education and Research, Chennai, IND; 2 General Medicine, Sri Ramachandra Institute of Higher Education and Research, Chennai, IND

**Keywords:** burkholderia pseudomallei, chronic infection, diabetes mellitus, melioidosis, splenectomy, splenic abscess

## Abstract

Melioidosis is a potentially fatal infection caused by Burkholderia pseudomallei, often presenting as various clinical manifestations and is commonly misdiagnosed in endemic regions, leading to delayed diagnosis. We report the case of a middle-aged female patient with uncontrolled diabetes mellitus and occupational exposure as a farmer, who presented with chronic fever and abdominal pain of one year duration. She had a prior history of a treated lumbar abscess where microbiological diagnosis was missed. Imaging revealed a multiloculated splenic abscess with features of impending rupture. Possible differentials of tuberculosis, infective endocarditis, and brucellosis were excluded through detailed evaluation. Splenectomy was performed due to the high risk of impending rupture. Microbiological analysis of splenic tissue confirmed B. pseudomallei. The patient was treated with intravenous meropenem followed by eradication therapy with cotrimoxazole and had a good clinical recovery. This case highlights the importance of considering melioidosis in chronic visceral abscess, especially in diabetic patients with environmental exposure.

## Introduction

Melioidosis, caused by Burkholderia pseudomallei, is an emerging tropical infection with diverse clinical manifestations [1,2]. It remains underdiagnosed in endemic regions due to its ability to mimic other chronic infections such as tuberculosis and brucellosis [1,3].

Diabetes mellitus is the most consistently identified predisposing factor, contributing to both susceptibility and disease severity [3]. In such patients, melioidosis may present insidiously, with chronic symptoms and atypical imaging findings, further contributing to diagnostic delays [1,3]. Among its various presentations, visceral abscess formation, particularly involving the spleen, is well described, although often misinterpreted during early evaluation [4]. Furthermore, B. pseudomallei was the most common etiological agent of splenic abscess in an area where melioidosis is endemic.

We present a case of chronic splenic abscess secondary to melioidosis with impending rupture, highlighting the diagnostic challenges and the importance of early clinical suspicion in high-risk individuals.

## Case presentation

A middle-aged female patient in her 50s, a known case of type 2 diabetes mellitus for 12 years with poor glycemic control, and a farmer by occupation, presented with complaints of intermittent fever and abdominal pain for one year. The abdominal pain was intermittent, pricking in nature, and localized to the left hypochondrium without clear aggravating and relieving factors. There was no associated history of anorexia, significant weight loss, vomiting or altered bowel habits. She had been evaluated at another hospital prior to presentation, where a CT scan of the abdomen revealed a splenic abscess (radiology images were unavailable with the patient), for which she received a short course of intravenous antibiotics (cephalosporins) for one week.

Additionally, she had a history of a left thigh skin abscess approximately 14 months prior, which was surgically drained. But the patient did not follow up on the details of the microbiological analysis of the pus culture. She was also recently diagnosed with an atrial septal defect (ASD) during routine evaluation.

On initial assessment, the patient was febrile (temperature 100°F). Blood pressure was 110/60 mmHg. Pulse rate was 98/min. Room air saturation was 100%. There was no generalized lymphadenopathy. Abdominal examination revealed tenderness in the left hypochondrium. There was no guarding or rigidity.

Laboratory parameters were as shown in Table 1.

**Table 1:** Basic blood investigations of the patient HbA1c: glycated hemoglobin; SGOT: serum glutamic oxaloacetic transaminase; SGPT: serum glutamic pyruvic transaminase.

Test/Laboratory parameter	Result	Reference range
Complete blood count		
Hemoglobin (g/dL)	12	13-17
Total leucocyte count (/mm^3^)	7,200	4,000-11,000
Platelet count (mm^3^)	2,00,000	150,000-450,000
Renal function tests		
Blood urea nitrogen (mg/dL)	9	7-20
Creatinine (mg/dL)	0.9	0.6-1.2
Liver function tests		
Aspartate aminotransferase (SGOT; U/L)	11	10-40
Alanine aminotransferase (SGPT; U/L)	17	7-56
Alkaline phosphatase (U/L)	61	44-167
Total protein (g/dL)	6.5	6.4-8.3
Albumin (g/dL)	3.8	3.5-5.0
Globulin (g/dL)	2.7	2.0-3.5
Total bilirubin (mg/dL)	0.1	0.2-1.2
Direct bilirubin (mg/dL)	0.03	0-0.3
Indirect bilirubin (mg/dL)	0.07	0.2-0.9
Diabetes profile		
HbA1c	11.3%	<5.7 % - Normal; <7% - Target in patients with diabetes
Inflammatory marker		
Erythrocyte sedimentation rate (ESR; mm/hr)	96	<20
Viral markers		
Hepatitis B	Non-reactive	-
Hepatitis C	Non-reactive	-
HIV	Non-reactive	-

**Figure 1:**
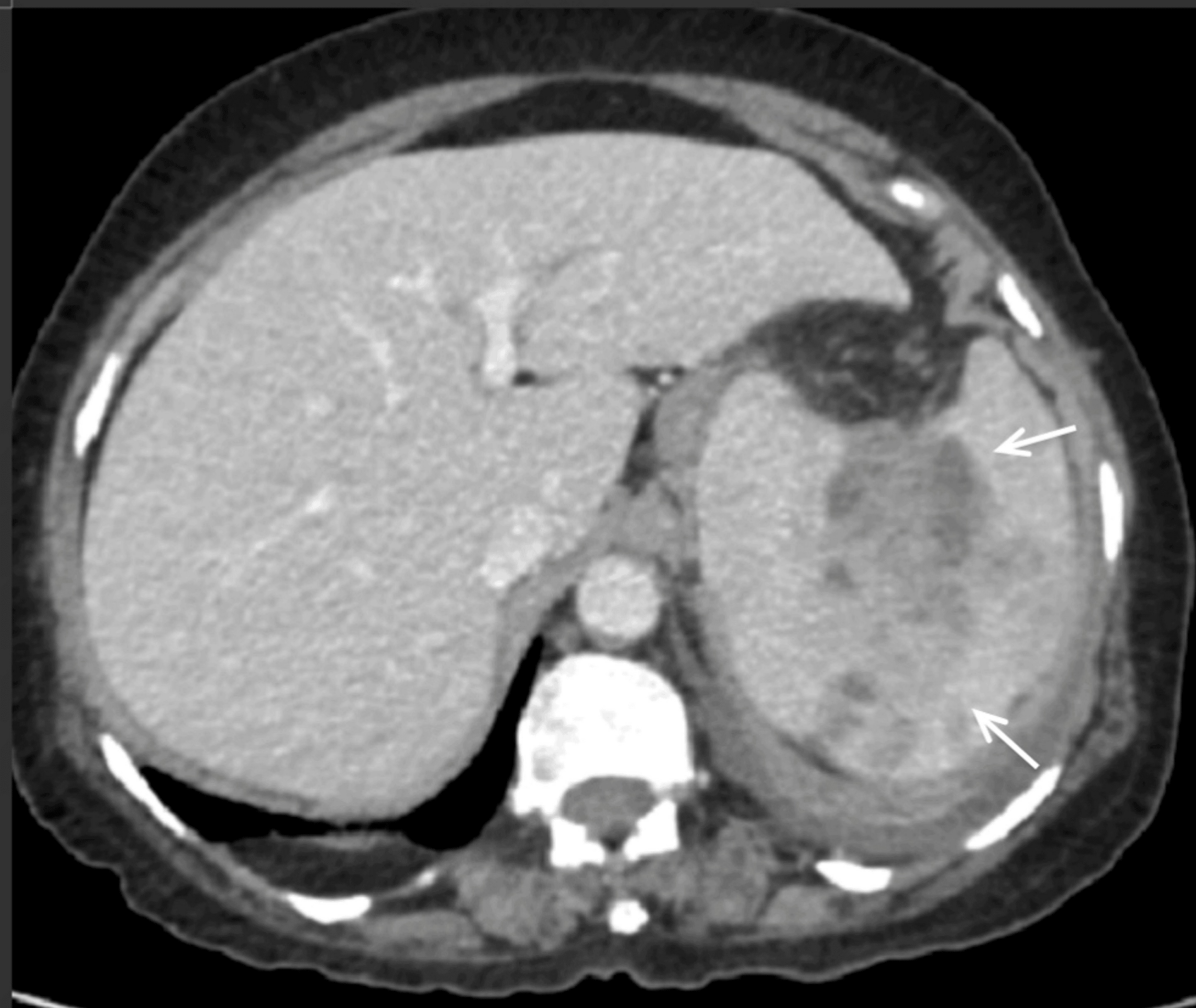
CECT abdomen showing multiple irregular peripherally enhancing multi locular collections within the splenic parenchyma CECT: Contrast-Enhanced Computed Tomography.

**Figure 2:**
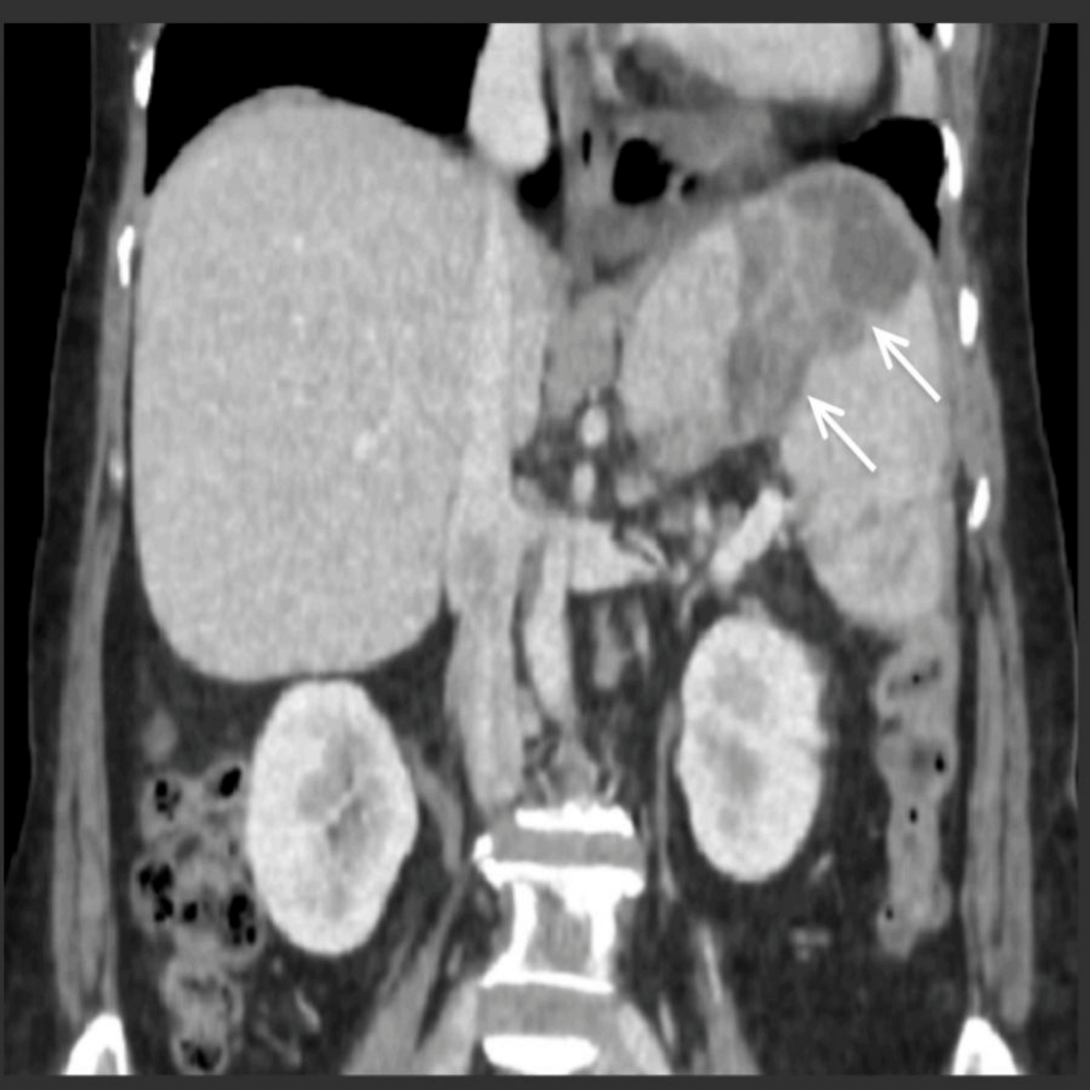
CECT abdomen showing extension of the splenic abscess into the sub-diaphragmatic region Capsular thinning and perisplenic inflammatory changes observed, which raised concern for impending rupture; Contrast-Enhanced Computed Tomography

Considering the evidence of splenic abscess, a contrast-enhanced CT (CECT) abdomen was done which showed multiple irregular peripherally enhancing multiloculated collections within the splenic parenchyma and extension of the abscess into the sub-diaphragmatic region with impending rupture (Figures 1, 2).

Based on the diffuse splenic abscess, differentials considered were tuberculosis (chronic course of illness and endemic area), melioidosis (uncontrolled diabetes and visceral abscess), brucellosis (occupational exposure), and infective endocarditis (underlying ASD).

**Figure 3:**
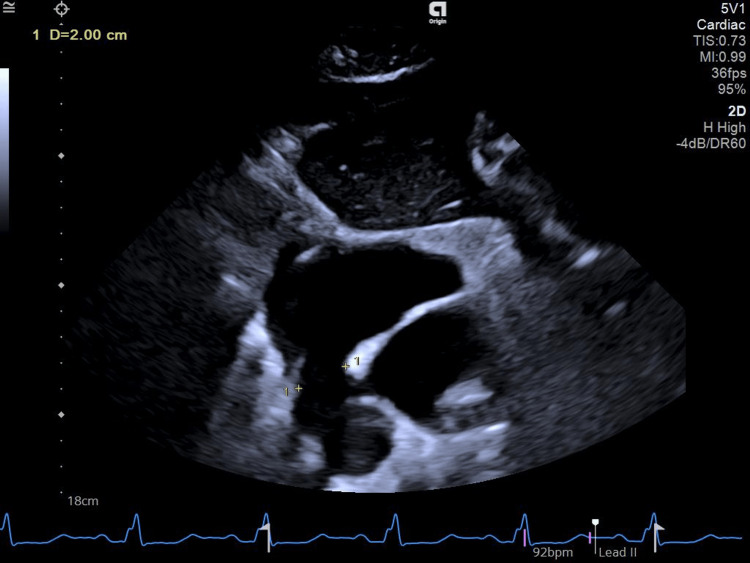
Transesophageal echo showing an atrial septal defect (ASD) of 2 cm

**Figure 4:**
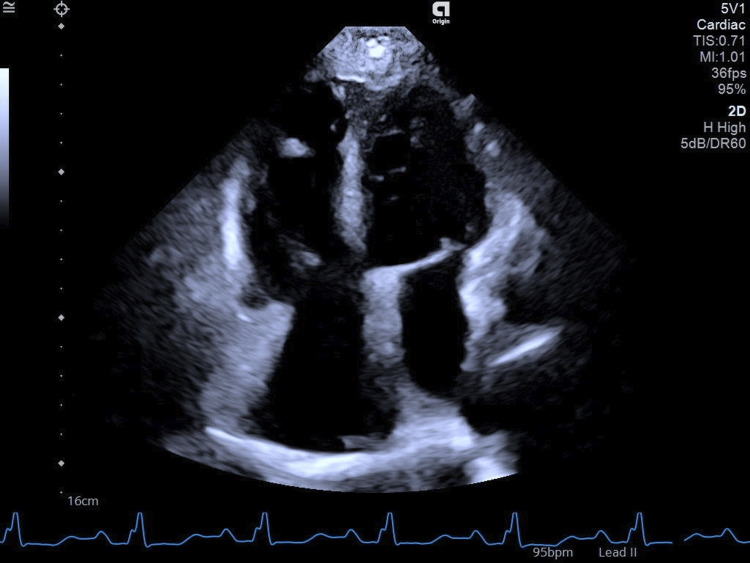
Transesophageal echo showing no vegetations over the mitral and tricuspid valves (ruling out infective endocarditis)

Two sets of blood cultures, aerobic and anaerobic, which were obtained before the initiation of empirical antibiotic therapy, were negative. Repeat blood cultures (two sets of aerobic and anaerobic) were also negative. Sputum analysis for acid-fast bacilli and Genexpert (Cepheid, Sunnyvale, California, USA) was negative. Brucella serology was also negative. Transesophageal echocardiography was performed, which revealed sinus venosus type ASD (20mm) with left-to-right shunt, no vegetations, and mild mitral regurgitation (Figures 3, 4).

Ultrasound-guided aspiration was attempted but could not be performed due to thick, solidified, and organized contents.

**Figure 5:**
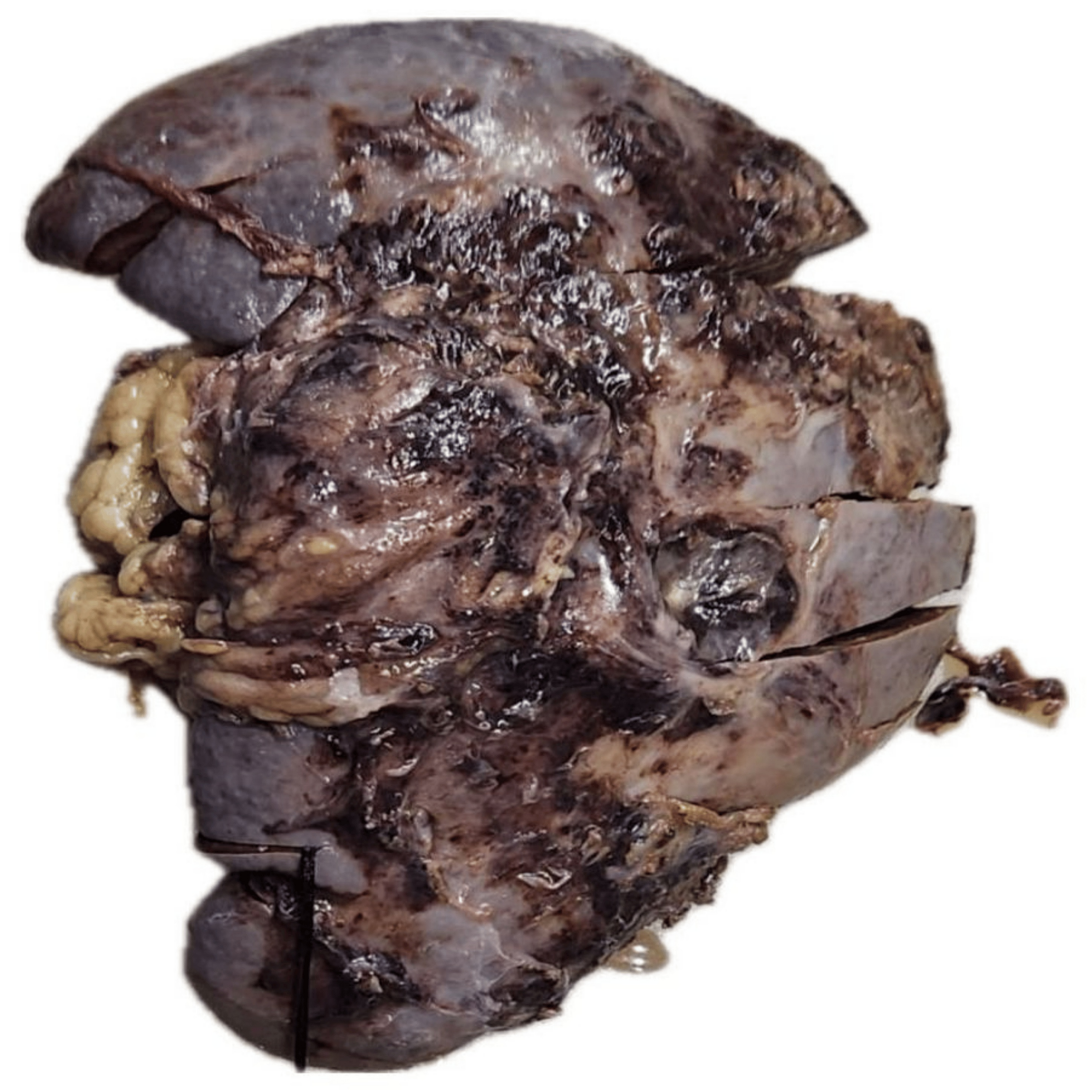
Splenectomy specimen showing multiple abscesses

Given the high risk of rupture, the patient underwent a splenectomy (Figure 5).

**Figure 6:**
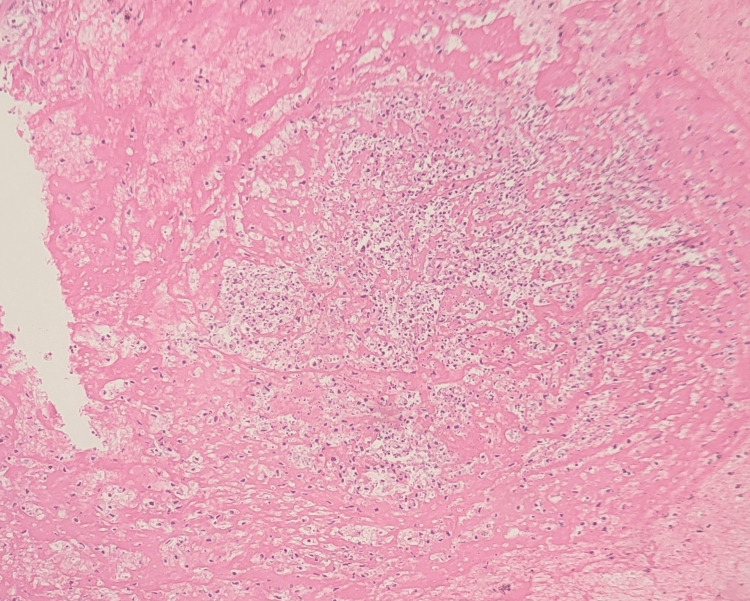
High power magnification of a hematoxylin and eosin stained section of the splenic tissue showing dense neutrophilic infiltration There is extensive tissue destruction with areas of necrosis, characterized by eosinophilic debris and loss of normal splenic sinusoidal pattern.

Intraoperative pus and tissue was sent for microbiological analysis. Gram stain revealed gram-negative bacilli with bipolar staining and histopathological examination showed dense neutrophilic infiltration (Figure 6).

Tissue culture sent from splenectomy on blood and macConkey agar yielded Burkholderia pseudomallei, identified by automated methods (Matrix-Assisted Laser Desorption/Ionization-Time of Flight or MALDI-TOF),with antimicrobial susceptibility testing performed as per Clinical and Laboratory Standards Institute (CLSI) guidelines [5] showing sensitivity to carbapenems and trimethoprim-sulfamethoxazole. The patient was diagnosed with melioidosis and was treated with intravenous meropenem (1 gm TID or three daily) and oral cotrimoxazole (double strength) for two weeks during the intensive phase. There was no further fever post splenectomy. She completed the intensive phase and was transitioned to eradication therapy.

During the one month follow-up, the patient remained clinically stable and was vaccinated against capsulated organisms. There was no recurrence of fever, and good glycemic control was achieved. She was continued on eradication therapy with cotrimoxazole. Review of prior medical records revealed that previous skin abscess (managed before the presentation to our center) had yielded B. pseudomallei on culture. This finding, on retrospective review, supported the diagnosis of disseminated melioidosis in the present case.

## Discussion

Melioidosis continues to pose a diagnostic challenge due to its heterogeneous clinical spectrum ranging from localized cutaneous infection to severe systemic disease, including pneumonia, visceral abscesses (liver, spleen, prostate), musculoskeletal involvement, genitourinary infection, neurological disease, and fulminant septicemia [1]. Splenic abscess, although a recognized manifestation, is often overlooked or attributed to alternative etiologies such as tuberculosis or infective endocarditis, particularly in endemic regions [1,2]. The present case underscores the classical association between uncontrolled diabetes and disseminated melioidosis. Diabetes is reported in a significant proportion of patients with visceral abscesses due to B. pseudomallei, likely reflecting impaired innate immune responses [3]. Additionally, occupational exposure, especially among agricultural workers, further increases the risk of acquisition [2].

One of the striking features in this case was the chronic, indolent course spanning several months, which closely mimicked other granulomatous or chronic infections. Similar atypical presentations have been documented, where melioidosis manifests as isolated or multiple splenic abscesses without overt systemic sepsis [4]. The prior history of inadequately treated cutaneous infection in our patient likely represents the initial portal of entry, followed by hematogenous dissemination. Such progression from localized to systemic disease has been reported in previous case series, emphasizing the importance of complete eradication therapy [3].

Radiologically, multiloculated abscesses involving the spleen, often with concurrent hepatic or psoas involvement, are characteristic but not pathognomonic [6]. In advanced cases, complications such as rupture or extension into adjacent structures may occur, necessitating surgical intervention, as seen in our patient [6]. Microbiological confirmation remains the gold standard for diagnosis. The identification of bipolar gram-negative bacilli and subsequent culture of B. pseudomallei are essential for definitive diagnosis and targeted therapy [1].

Management of melioidosis requires a two-phase approach consisting of an intensive phase with intravenous antibiotics such as meropenem or ceftazidime, followed by a prolonged eradication phase using oral cotrimoxazole [1,7]. Failure to complete the eradication phase is associated with a high risk of relapse, which may explain the recurrence and dissemination observed in some patients [8,9].

## Conclusions

Melioidosis remains an under-recognized infection in many endemic regions due to its diverse clinical presentations and its ability to closely mimic other chronic infectious conditions. This often results in delayed diagnosis, particularly in patients presenting with indolent symptoms or atypical imaging findings. In our case, the prolonged clinical course and overlapping features with more common conditions, such as tuberculosis, highlight the diagnostic challenges associated with this disease. A high index of suspicion is therefore essential, especially in individuals with established risk factors such as uncontrolled diabetes mellitus and occupational exposure.

This case also emphasizes the importance of a systematic diagnostic approach combining clinical evaluation, radiological findings, and microbiological confirmation. Early identification of B. pseudomallei is crucial, as timely initiation of appropriate antimicrobial therapy significantly influences patient outcomes. In advanced cases, particularly when complications such as impending rupture are present, surgical intervention may be necessary as part of definitive management.

Furthermore, adherence to the recommended two-phase treatment strategy-comprising an intensive phase followed by prolonged eradication therapy-is critical to prevent relapse and ensure complete disease resolution. The history of prior inadequately treated infection in our patient further underscores the importance of appropriate initial management and follow-up. Increased awareness among clinicians regarding the varied presentations of melioidosis can aid in early recognition and reduce associated morbidity and mortality.
